# Genetic Analysis Using a Next Generation Sequencing-Based Gene Panel in Patients With Skeletal Dysplasia: A Single-Center Experience

**DOI:** 10.3389/fgene.2021.670608

**Published:** 2021-05-26

**Authors:** Su Jin Kim, Sae-Mi Lee, Jong-Moon Choi, Ja-Hyun Jang, Hyun Gi Kim, Jung-Taek Kim, Jae Ho Cho, Young Bae Sohn

**Affiliations:** ^1^Department of Pediatrics, Inha University Hospital, Inha University College of Medicine, Incheon, South Korea; ^2^GC Genome, GC Labs, Yongin-si, South Korea; ^3^Department of Laboratory Medicine, Kangwon National University School of Medicine, Chuncheon-si, South Korea; ^4^Department of Laboratory Medicine and Genetics, Samsung Medical Center, Seoul, South Korea; ^5^Department of Radiology, Eunpyeong St. Mary’s Hospital, College of Medicine, The Catholic University of Korea, Seoul, South Korea; ^6^Department of Orthopedic Surgery, Ajou University Hospital, Ajou University School of Medicine, Suwon-si, South Korea; ^7^Department of Medical Genetics, Ajou University Hospital, Ajou University School of Medicine, Suwon-si, South Korea

**Keywords:** skeletal dysplasia, next-generation sequencing, genetic heterogeneity, skeletal genetics, molecular genetic test

## Abstract

Skeletal dysplasia (SD), a heterogeneous disease group with rare incidence and various clinical manifestations, is associated with multiple causative genes. For clinicians, accurate diagnosis of SD is clinically and genetically difficult. The development of next-generation sequencing (NGS) has substantially aided in the genetic diagnosis of SD. In this study, we conducted a targeted NGS of 437 genes – included in the nosology of SD published in 2019 – in 31 patients with a suspected SD. The clinical and genetic diagnoses were confirmed in 16 out of the 31 patients, and the diagnostic yield was 51.9%. In these patients, 18 pathogenic variants were found in 13 genes (*COL2A1, MYH3, COMP, MATN3, CTSK, EBP, CLCN7, COL1A2, EXT1, TGFBR1, SMAD3, FIG4*, and *ARID1B*), of which, four were novel variants. The diagnosis rate was very high in patients with a suspected familial SD and with radiological evidence indicating clinical SD (11 out of 15, 73.3%). In patients with skeletal involvement and other clinical manifestations including dysmorphism or multiple congenital anomalies, and various degrees of developmental delay/intellectual disability, the diagnosis rate was low (5 out of 16, 31.2%) but rare syndromic SD could be diagnosed. In conclusion, NGS-based gene panel sequencing can be helpful in diagnosing SD which has clinical and genetic heterogeneity. To increase the diagnostic yield of suspected SD patients, it is important to categorize patients based on the clinical features, family history, and radiographic evidence.

## Introduction

Skeletal dysplasia (SD) is a group of heterogeneous genetic disorders associated with bone and cartilage development. It is characterized by orthopedic symptoms such as short stature, limb or spine deformities, and precocious osteoarthritis, and is sometimes accompanied by pulmonary, genitourinary, visual, auditory, and neurodevelopmental manifestations (Bae et al., 2016; Maddirevula et al., 2018; Uttarilli et al., 2019). In 2019, the International Skeletal Dysplasia Society published an updated version of the nosology of SD comprising 461 genetic skeletal disorders and 437 different causative genes classified into 42 groups based on their clinical, radiographic, and/or molecular phenotypes (Mortier et al., 2019). The clinical diagnosis of SD can be difficult because of its relatively rare incidence; for instance, the incidence rate of osteogenesis imperfecta (OI), which is the most common disease entity of SD, is 1 in 15,000 live births worldwide (Fratzl-Zelman et al., 2015). The varying and non-specific clinical symptoms among SD, especially radiological findings requiring interpretation by experts, constitute another factor hindering clinical diagnosis (Zankl et al., 2007; Calder, 2020). Molecular diagnosis is also complex because variants of one gene can cause different phenotypes, whereas similar clinical manifestations can result from variants of different genes. For example, molecular defects in *FGFR3* (MIM#134934), which encodes fibroblast growth factor receptor 3, can lead to various disease spectra of SD. It can present with lethal thanatophoric dysplasia type 1, achondroplasia, craniosynostosis, or other *FGFR3*-related disorders such as lacrimo-auriculo-dento-digital (LADD) syndrome. On the contrary, OI type 3 (progressively deforming type) can result from mutations in multiple genes including I*FITM5, SERPINF1, BMP1*, in addition to representative genes such as *COL1A1* and *COL1A2* (Mortier et al., 2019). For these reasons, determining the gene tests to be performed on patients with a suspected SD is challenging to clinicians. In recent years, next-generation sequencing (NGS) technology, including whole exome sequencing (WES) and whole genome sequencing (WGS), has been a helpful tool for clinicians to diagnose SD (Lazarus et al., 2014; Zhang et al., 2015; Bae et al., 2016). In this study, we performed a targeted NGS in 31 patients with a suspected SD during a 3-year period at a single tertiary center in South Korea and analyzed their clinical and molecular genetic data.

## Patients and Methods

### Patients and Clinical Evaluation

This study was approved by the Institutional Review Board (AJIRB-BMR-GEN-20-519) of Ajou University Hospital, and all participants and guardians of the pediatric patients provided an informed consent. From March 2017 to February 2020, patients were enrolled in this study when they met one or more of the following criteria: (1) suspected familial SD, (2) radiological abnormalities suggesting SD with or without a family history, and (3) skeletal manifestations with developmental delay (DD)/intellectual disability (ID), or multiple congenital anomalies (MCA), with or without a family history. The exclusion criteria were as follows: known chromosomal aberrations or patients with a confirmed diagnosis by Sanger sequencing due to their characteristic clinical features such as achondroplasia. The patients and available family members were examined by clinical geneticists for detailed clinical phenotypes including past medical histories through a review of medical records, pedigree analyses, physical examination, and laboratory tests. Radiological skeletal surveys were performed by an expert radiologist.

### Targeted NGS Panel and Genetic Analysis

Genomic DNA was extracted from peripheral blood. A library was prepared using the xGEN^®^ Inherited Panel (Integrated DNA Technologies, Coralville, IA, United States), which comprised 5,000 genes and 180 SNP sites. Among the 5,000 genes, we analyzed 437 genes listed in the nosology of skeletal dysplasia published in 2019 (Mortier et al., 2019). Massively parallel sequencing was performed using the Illumina NextSeq 500^®^ sequencing platform (Illumina Inc., San Diego, CA, United States). The mean coverage of target regions was 143x, with 99% of bases covered by at least 10 reads. Sequence reads were aligned to hg19 using the Burrow-Wheeler Aligner (version 0.7.12, MEM algorithm). Duplicate reads were removed using Picard-tools version 1.96. Local realignment, base quality score recalibration, and variant calling were performed using the Genome Analysis Toolkit (GATK version 3.5). Variants were annotated using the Variant Effect Predictor and dbNSFP. The identified variants were confirmed by Sanger sequencing. Copy number variants were called from the panel data using XHMM which is based on a depth–based method (Fromer et al., 2012). Familial segregation tests were performed by Sanger sequencing for the indicated cases. Variants with minor allele frequencies over 1% in the 1,000 genomes browser^1^, NHLBI ESP Exome Variant Server^2^, genome Aggregation Database (gnomAD)^3^, and Korean Reference Genome Database (KRGDB)^4^ were excluded. For the *in silico* analysis of missense variants, the Sorting Intolerant From Tolerant (SIFT)^5^, PolyPhen-2^6^, and Mutation Taster^7^ algorithms were used to predict the variants to damage protein function. The identified variants were classified according to the guidelines for the interpretation of sequence variants by the American College of Medical Genetics and Genomics and Association for Molecular Pathology (Richards et al., 2015). All sequences generated for this project have been submitted to the NCBI SRA (BioProject ID PRJNA718975).

## Results

### Demographics and Clinical Manifestations of Patients

In total, 31 Korean patients (11 females and 20 males) from 31 non-consanguineous families, were included in this study. The patients were divided into six categories according to their clinical manifestations and family history (Table 1). The detailed demographics and clinical manifestations of the enrolled patients are summarized in Table 2. The median age of the patients was 10.0 years (range, 1 month to 46 years) and the median height standard deviation score (SDS) was −0.95 (range, −4.6 to 2.7). The most common clinical symptoms were familial or non-familial short stature (7/31, 22.5%) and abnormalities of the spine such as scoliosis and kyphosis (7/31, 22.5%) (Table 2).

**TABLE 1:** Categories of the patients.

Categories	Suspected of family SD	Radiologic abnormalities	Various phenotypes with skeletal involvement			
			Dysmorphism/multiple congenital anomalies	Mild DD/ID	Moderate DD/ID	Severe DD/ID
1 (*n* = 7)	Yes	Yes				
2 (*n* = 5)		Yes				
3 (*n* = 3)	Yes		Yes			
4 (*n* = 5)			Yes	Yes		
5 (*n* = 9)			Yes		Yes	
6 (*n* = 2)			Yes			Yes

*SD, skeletal dysplasia; DD, developmental delay; ID, intellectual disability.*

**TABLE 2:** Demographics and clinical manifestations of the patients.

Categories	Case (#)	Sex	Age	Height (cm)	Height-SDS	Weight (Kg)	Weight-SDS	Phenotypes
**1**	1	M	1 month	52	–0.9	4.8	0.3	familial short stature (affected mother), frontal bossing, short femur in prenatal ultrasonography, horizontal acetabuli of the pelvic bone (Figure 1A)
	2	F	1 month	49	–1.5	4.2	0	familial SD (affected mother), mild flaring of the metaphysis of long bone, not yet ossified epiphysis
	3	F	43 years	125	–4.6	50	–0.3	familial short stature (affected father), bilateral dysplastic hips, bilateral destructed femoral head, deformity of rib cage showing fanning and inward bowing (Figure 1B).
	4	M	20 years	173.2	–0.1	66.1	–0.1	familial SD (affected father), anterior wedging of spine, flat acetabulum
	5	F	17 years	143	–2.2	41	–1.2	familial SD (affected mother), severe scoliosis, bowing of humerus, dysplasia of hip (Figure 1C)
	6	M	19 years	160.8	–1.6	54.5	–1.0	familial SD (affected father and sister), multiple exostosis (Figure 1D)
	7	M	16 years	146.8	–3.0	48.9	–1.1	familial SD, short stature (maternal uncle), platyspondyly, scoliosis, epiphyseal irregularity

**2**	8	M	10 years	128.1	–1.4	28.1	–0.8	short stature, metaepiphyseal irregularity of knee (Figure 1E)
	9	F	40 years	161	0.1	30	–2.1	frontal bossing, increased cortical bone thickness, BMD L1-4 spine T score 3.5, femur T score 5.2
	10	M	46 years	175	0.1	83	1.0	increased cortical bone thickness, BMD L1-4 spine T score 3.8, femur T score 5.3 (Figure 1F)
	11	M	3 years	92.3	–1.2	14.2	–0.6	dentinogenesis imperfecta
	12	F	13 years	143.6	–1.6	56.5	0.6	disproportional short stature, cubitus valgus, short forearms, epi-metaphyseal dysplasia, mild anterior spinal wedging

**3**	13	M	16 years	176.7	0.5	52.5	–0.9	familial SD (affected mother), tall and thin, hypertelorism, mild scoliosis, both flat feet (Figure 2)
	14	M	6 years	132.5	1.9	26.3	0.7	familial tall stature (affected mother), lens subluxation, pectus carinatum
	15	F	29 years	146	–1.9	44	–0.9	familial short stature (affected daughter), deafness, ptosis, cubitus valgus

**4**	16	M	19 months	78.8	–1.0	10.3	–0.4	facial asymmetry, torticollis, short neck, multiple spinal fusion (Figure 3)
	17	M	3 years	103.9	0.6	14.2	–0.6	surgical repair of inguinal hernia, cleft palate, scoliosis, clinodactyly, flat feet, arachnodactyly, pectus carinatum
	18	M	7 years	126.7	0.2	34.8	1.3	macrocephaly, strabismus, ventricular septal defect, scoliosis, genu valgum
	19	F	19 years	145.7	–2.0	36.5	–1.6	short stature, panhypopituitarism, deafness, ataxia
	20	F	3 years	94.2	–0.7	12.8	–0.9	facial dysmorphism, limitation of eyeball movement, scoliosis

**5**	21	M	17 months	80.5	–0.6	10.6	–0.3	coarse, puffy face, hirsutism, scoliosis and kyphosis, atrial septal defect
	22	F	18 years	141.2	–2.6	52.1	–0.2	short stature, coarse face, high arched palate, scoliosis, and kyphosis
	23	F	4 months	51	–3.3	4.7	–1.3	short stature, coarse face, depressed nasal bridge, umbilical hernia, failure to thrive, hirsutism, hypothyroidism
	24	M	10 years	156.1	1.5	69.9	–0.8	arthrogryposis (surgically corrected), strabismus, obesity
	25	M	14 months	73.3	–1.2	8.4	–1.0	failure to thrive, strabismus, broad nasal bridge, low set ears, hip dysplasia
	26	M	15 years	149.9	–2.2	64.1	0.2	severe scoliosis and arthrogryposis (surgically corrected), infantile hypotonia
	27	M	38 years	174	–0.1	50	–1.3	microcephaly, facial dysmorphism, deafness, scoliosis
	28	F	5 years	108.5	–0.4	20.7	0.3	facial dysmorphism, scoliosis, kyphosis
	29	M	7 years	125.3	0	38.2	1.8	macrocephaly, facial dysmorphism, scoliosis, genu valgum

**6**	30	M	5 months	63.5	–0.7	6.4	–0.8	severe hypotonia, failure to thrive, optic dystrophy, nystagmus, thinning of corpus callosum, hypospadias, hypoplasia of finger and toes, dysplasia of hip **(Figure 4)**
	31	M	15 months	88.3	2.7	11	0.5	brachycephaly, down slanted palpebral fissure, ptosis, blephalophimosis, bilateral 3,4th finger syndactyly, campylodactyly

*SDS, standard deviation score; SD, skeletal dysplasia. BMD, bone mineral density.*

### Molecular Diagnosis of Patients

Next-generation sequencing-based targeted gene analysis and further familial segregation studies identified 13 causative genes in 16 out of a total of 31 patients (51.6% diagnostic yield). The genetic variants of these 16 patients with confirmed clinical and genetic diagnoses are summarized in Table 3. Eighteen pathogenic or likely pathogenic variants in thirteen genes were identified, of which, four were novel (Table 4). The identified genes and final diagnoses of the patients were heterogeneous as follows; two type II collagenopathy including spondylometaphyseal dysplasia congenita and spondyloepiphyseal dysplasia (***COL2A1***, collagen type II, alpha-1), spondylocarpotarsal synostosis syndrome (***MYH3***, myosin heavy chain 3), two multiple epiphyseal dysplasia (***COMP***, cartilage oligomeric matrix protein and ***MATN3***, matrilin-3), pseudoachondroplasia (***COMP***, cartilage oligomeric matrix protein), pycnodysostosis (***CTSK*,** lysosomal cysteine protease, cathepsin K), chondrodysplasia punctata (***EBP***, emopamil-binding protein), osteopetrosis (***CLCN7***, chloride channel 7), osteogenesis imperfecta (***COL1A2*,** collagen type I, alpha-2), multiple cartilaginous exostoses (***EXT1***, exostosin glycosyltransferase 1), Loeys-Dietz syndrome type 1 (***TGFBR1***, transforming growth factor- β receptor, type 1), Loeys-Dietz syndrome type 3 (***SMAD3*,** SMAD family member 3), Yunis-Varon dysplasia (***FIG4***, phosphoinositide 5-phosphatase), and two cases of Coffin-Siris syndrome (***ARID1B***, at-rich interaction domain-containing protein 1B). One gene (***EBP)*** was inherited in an X-linked dominant manner, two genes (***CTSK, FIG4***) were inherited in an autosomal recessive manner, and the remaining 10 genetic variants were inherited in an autosomal dominant manner.

**TABLE 3:** Genetic variants of 16 patients with a confirmed diagnosis’.

	#	Gene	Transcript	Nucleotide change	Amino acid change	Zygosity	Inheritance	ACMG classification (evidence)	Novelty	Final diagnosis
**1**	1	*COL2A1*	NM_001844.4	c.2059G > A	p.Gly687Ser	Het	AD	LPV (PM1, PM2, PP1, PP3, PP4, PP5)	PMID 25967556	spondyloepiphyseal dysplasia congenita
	2	*COL2A1*	NM_001844.4	c.2756C > T	p.Pro919Leu	Het	AD	LPV (PM1, PP1, PP3, PP4, PP5)	this study	spondyloepiphyseal dysplasia (Namaqualand type)
	3	*COMP*	NM_000095.2	c.1417_1419del	p.Asp473del	Het	AD	PV (PS3, PM1, PM2, PM4, PM6, PP4, PP5)	PMID 7670471	Pseudoachondroplasia
	4	*COMP*	NM_000095.2	c.1519G > A	p.Asp507Asn	Het	AD	LPV (PM1, PM2, PP3, PP4)	PMID 21965141	Multiple epiphyseal dysplasia
	5	*EBP*	NM_006579.2	c.246G > T	p.Trp82Cys	Het	XD	LPV (PM1, PM2, PP3, PP4)	PMID 12483303	X-linked dominant chondrodysplasia punctata
	6	*EXT1*	NM_000127.2	c.444delC	p.Ser149Alafs*8	Het	AD	PV (PVS1, PM2, PP4)	this study	Multiple cartilaginous exostoses
**2**	8	*MATN3*	NM_002381.5	c.361C > T	p.Arg121Trp	Het	AD	PV (PS2,PM1, PM2, PP3, PP4, PP5)	PMID 11479597	Multiple epiphyseal dysplasia
	9	*CTSK*	NM_000396.3	c.755G > A	p.Ser252Asn	Comp. Het	AR	LPV (PM2, PM3, PP3, PP4)	PMID 28328823	Pycnodysostosis
				c.426delT	p.Phe142Leufs*19			PV (PVS1, PM2, PP4, PP5)	PMID 10634420	
	10	*CLCN7*	NM_001287.6	c.2284C > T	p.Arg762Trp	Het	AD	LPV (PM1, PM5, PP3, PP4)	PMID 19953639	Osteopetrosis
	11	*COL1A2*	NM_000089.3	c.1801G > A	p.Gly601Ser	Het	AD	LPV (PM1, PM2, PM6, PP3, PP4, PP5)	PMID 11317364, 26177859	Osteogenesis imperfecta
**3**	13	*SMAD3*	NM_005902.3	c.1267A > G	p.Ser423Arg	Het	AD	LPV (PM1, PM2, PM5, PP1, PP3, PP4)	PMID 24804794	Loeys-Dietz syndrome type 3
**4**	16	*MYH3*	NM_002470.3	c.1581 + 2T > C		Het	AD	PV (PVS1. PM2, PP4)	this study	Spondylocarpotarsal fusion syndrome 1A,
	17	*TGFBR1*	NM_004612.2	c.1120G > A	p.Gly374Arg	Het	AD	LPV (PM2, PM6, PP3, PP4)	PMID 16928994	Loeys-Dietz syndrome 1
**5**	21	*ARID1B*	NM_020732.3	c.3223C > T	p.Arg1075*	Het	AD	PV (PVS1, PM2, PP4, PP5)	PMID 22426309	Coffin–Siris syndrome
	22	*ARID1B*	NM_020732.3	c.4251delG	p.Arg1417Serfs*31	Het	AD	PV (PVS1, PM2, PP4)	this study	Coffin–Siris syndrome
**6**	30	*FIG4*	NM_014845.5	c.506A > C	p.Tyr169Ser	Comp. Het	AR	LPV (PM2, PM3, PP3, PP4)	PMID 32385905	Yunis-Varon syndrome
				c.1750 + 1del				PV (PVS1, PM2, PP4, PP5)	PMID 24088667	

*ACMG, American College of Medical Genetics and Genomics; Het, heterozygote; AD, autosomal dominant; LPV, likely pathogenic variant; PM, pathogenic moderate; PP, pathogenic supporting; PS, pathogenic strong; PVS, pathogenic very strong; PV, pathologic variant; PMID, pubmed identifier; XD, X-linked dominant; Comp. Het, compound heterozygotes; AR, autosomal recessive.*

**TABLE 4:** Novel variants of four patients with a confirmed diagnosis.

Case #	Gene	Transcript	Nucleotide change	Amino acid change (type of variant)	*In silico* prediction (SIFT, PolyPhen-2, Mutation Taster)	Maximum allele frequency in GenomeAsia 100k population, KRGDB	ACMG evidence	ACMG classification
**2**	*COL2A1*	NM_001844.4	c.2756C > T	p.Pro919Leu (missense variant)	disease causing	absent	PM1, PP1, PP3, PP4, PP5	Likely pathogenic
**6**	*EXT1*	NM_000127.2	c.444delC	p.Ser149Alafs*8 (Null variant:frameshift)		absent	PVS1, PM2, PP4	Pathogenic
**16**	*MYH3*	NM_002470.3	c.1581 + 2T > C	(Null variant; canonical splice site)		absent	PVS1, PM2, PP4	Pathogenic
**22**	*ARID1B*	NM_020732.3	c.4251delG	p.Arg1417Serfs*31 (Null variant:frameshift)		absent	PVS1, PM2, PP4	Pathogenic

*ACMG, American College of Medical Genetics and Genomics; SIFT, Sorting Intolerant From Tolerant; KRGDB, Korean Reference Genome Database.*

### Representative Cases With a Confirmed Molecular Genetic Diagnosis

**FIGURE 1:**
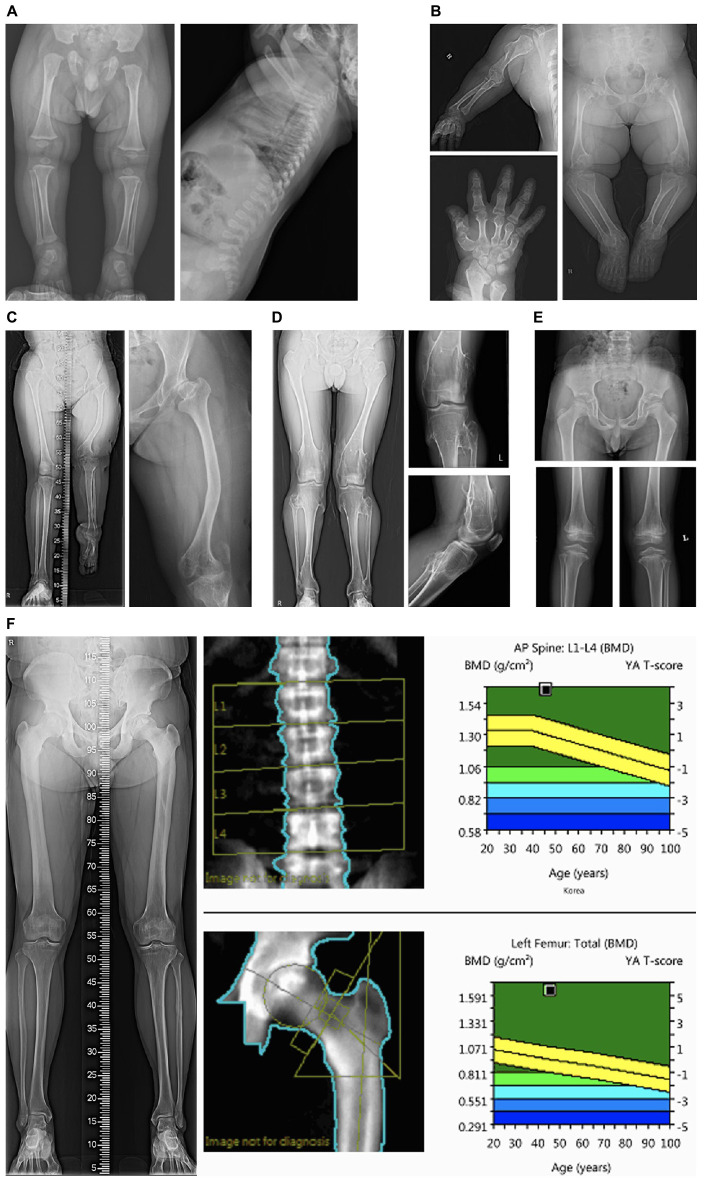
Representative skeletal survey images of patients in categories 1 and 2. **(A)** Radiographic images of case #1 with type II collagenopathy (spondyloepiphyseal dysplasia congenita) showing horizontal acetabuli of the pelvic bone and a pear-shaped lumbar vertebral body at 8 months of age; **(B)** Case #3 with pseudoachondroplasia showing dysplastic and relatively short upper and lower extremity bones. Bilateral femur heads are flattened and both acetabuli show degenerative changes; **(C)** case #5 with X-linked dominant chondrodysplasia punctata showing shortening and bowing of the left femur, dysplastic hips; **(D)** case #6 with multiple cartilaginous exostoses showing multiple exostoses in the bilateral distal femur and proximal tibia; **(E)** case #8 with multiple epiphyseal dysplasia showing small and dysplastic both proximal femoral epiphyses and acetabular dysplasia. Both knees show epiphyseal and metaphyseal irregularity; **(F)** Case #10 with osteopetrosis showing increased cortical bone thickness. The bone densitometry T-scores of the spine and femur were 3.5 and 5.2, respectively.

The patients in categories 1 and 2 who had radiological abnormalities suggesting SD had a higher diagnostic rate (85.7% and 100%, respectively) than those in the other categories. Figures 1A–F shows the representative radiological findings of patients in categories 1 and 2 with confirmative molecular diagnoses. The frequently observed abnormal findings in the skeletal surveys included shortening and metaphyseal or epiphyseal dysplasia of long bones, dysplastic hips, and dysplasia of the vertebrae. Abnormal bone densitometry (BMD) suggested osteoporotic or osteosclerotic genetic metabolic bone diseases. In category 2, case 9 who was diagnosed with pycnodysostosis and reported previously by us, had an atypical mild presentation with normal height and recurrent fractures (Song et al., 2017). Case 10 had an increased cortical bone thickness in the skeletal survey, and the BMD T scores of the spine and femur were 3.5 and 5.2, respectively. Case 11 presented with a severe dentinogenesis imperfecta only. After the identification of the likely pathogenic variants in *COL1A2*, BMD surveillance revealed that the BMD of the total body less the head was 0.439 g/cm^2^ (a normal reference for BMD under the age of 5 was not available). Considering his young age, the definition of the OI type should take into account the clinical symptoms that appear as he grows. Regular bisphosphonate infusion could be started after the molecular diagnosis of OI before he suffered a serious bone fracture.

**FIGURE 2:**
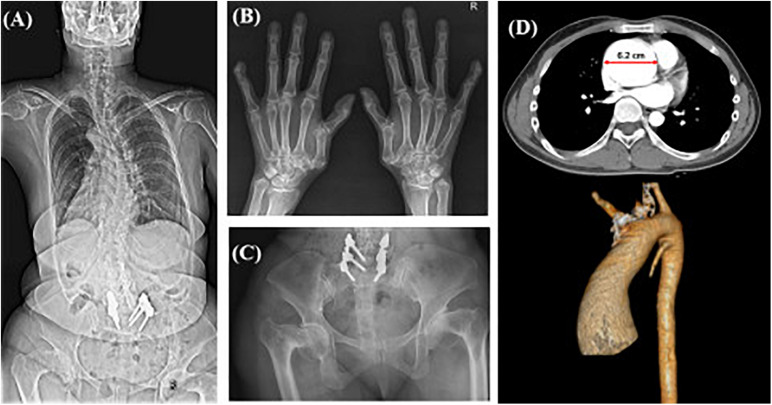
Radiographic images of case #13 and affected mother with aneurysms-osteoarthritis syndrome. X-ray images of the patient’s affected mother showing **(A)** severe scoliosis despite surgical correction, **(B)** degenerative osteoarthritis of the hand joints with subluxation of the 1st carpometacarpal joints, **(C)** bilateral severe coxa vara deformities; **(D)** CT scan with 3D reconstruction of Case #13 showing aneurysm of the ascending aorta with a dilated root (diameter: 6.2 cm).

Case 13 in category 3 had clinical manifestations similar to those of his mother including facial dysmorphism (hypertelorism, dolichocephaly), tall height, and mild scoliosis. His mother had a severe scoliosis and osteoarthritis since her 40’s (Figures 2A–C). Although his skeletal symptoms were not evident, this patient underwent a targeted NGS because of a suspected familial SD and was diagnosed with Loeys-Dietz syndrome type 3 (aneurysms-osteoarthritis syndrome) caused by a pathologic variant in *SMAD3*, which was inherited from his mother. After diagnosis, he underwent CT angiography for the evaluation of an aneurysm, which revealed a severely dilated root of the ascending aorta (Figure 2D). Bentall’s operation for the aortic aneurysm and severe aortic regurgitation was performed, which enabled the patient to avoid sudden critical vascular events.

**FIGURE 3:**
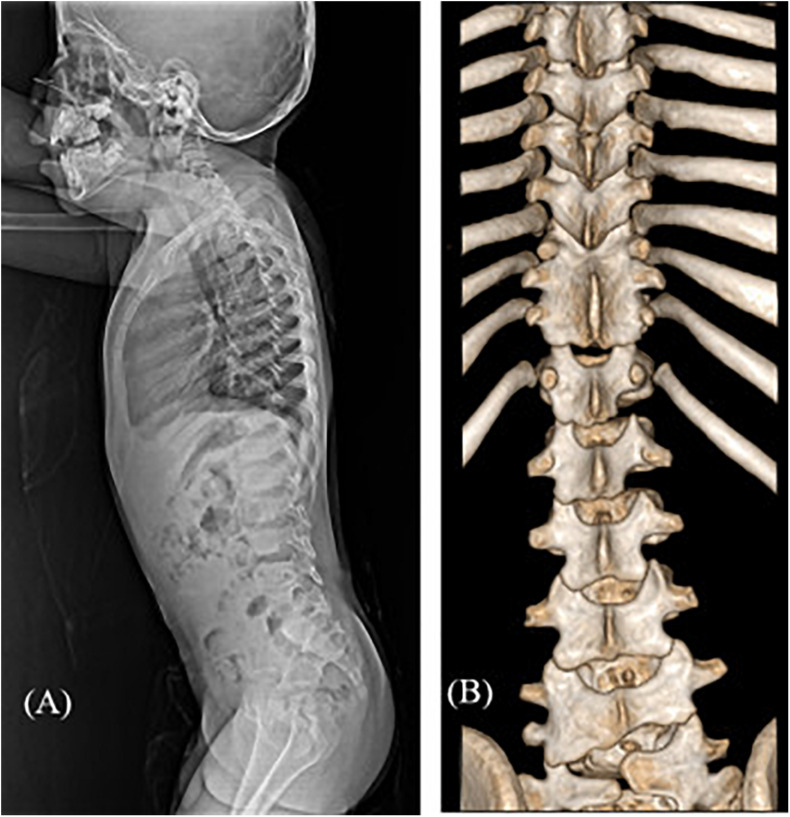
Radiographic images of case #16 with spondylocarpotarsal fusion syndrome 1A. **(A)** Lateral spine X-ray showing mildly narrowed disk spaces **(B)** 3D reconstruction CT scan showing fusion of T9-10-11 bilateral facet joints and spinous processes and L4-5 left facet joints.

Among the 16 patients in categories 4–6, five patients were diagnosed with SD (31.3% diagnostic yield) (Table 3). Case 16 presented a mild developmental delay, an asymmetric face, torticollis, a short neck, and scoliosis. X-ray and CT scans of the spine revealed narrowing of the disk space and multiple fusions of the spinal processes (Figures 3A,B). Two patients (cases 21 and 22) with skeletal manifestations such as scoliosis and kyphosis and moderate developmental delay were diagnosed with *ARID1B*-related Coffin-Siris syndrome. Genetic counseling and early intervention with special education could be initiated, especially in younger patients (17 months of age) after confirmative diagnosis.

**FIGURE 4:**
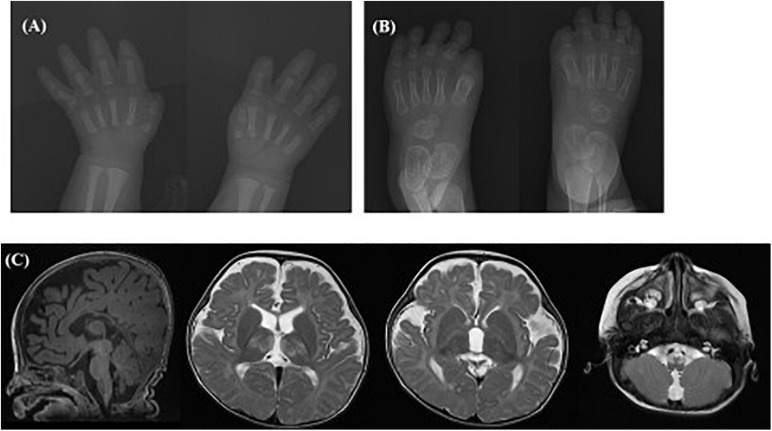
Radiographic images of case #30 with Yunis-Varon dysplasia. X-ray showing hypoplasia and agenesis of the distal phalanx of the **(A)** fingers and **(B)** toes. **(C)** Brain MRI showing diffuse corpus callosal thinning, prominent subarachnoidal space at the frontotemporal convexity, and dilation of the 3rd ventricle, T2 high signal intensity of the bilateral thalami and medulla oblongata of inferior olivary nuclei.

To our knowledge, case 30 in category 6 was the first case diagnosed with Yunis-Varon dysplasia in South Korea. He presented MCA including hypopigmented skin and hair, facial dysmorphism, ocular symptoms (optic nerve dystrophy, nystagmus), hypoplasia, and dysplastic distal phalanx of the fingers and toes (Figures 4A,B), developmental dysplasia of hip (DDH), hypospadias, and a bifid scrotum. The patient suffered respiratory difficulty with laryngomalacia, severe developmental delay with muscular hypotonia, and failure to thrive. A brain MRI taken at 8 months of age showed structural brain anomalies including diffuse corpus callosal thinning, a prominent subarachnoid space at the frontotemporal convexity, dilation of the 3rd ventricle, and T2 high signal intensity of the bilateral medulla oblongata in the inferior olivary nuclei (Figure 4C). In the targeted NGS, compound heterozygous variants of the *FIG4* gene were identified (Table 3) in the patient, and his parents were heterozygous carriers. His developmental milestone did not progress at all; he could not control his head completely or establish eye contact until 15 months of age. He died at of 15 months of age due to respiratory failure.

## Discussion

This study explored the diagnoses of various spectrums of SD through a targeted NGS in 31 patients with a suspected SD at a single tertiary center in South Korea over a 3-year period. In this study, we could make a definite diagnosis in 16 patients based on the clinical and molecular findings using NGS. In total, 18 pathogenic/likely pathogenic variants of 13 different genes were found in 16 patients, out of which, 10 genes were found in one patient each, and three genes (*COL2A1, COMP*, and *ARID1B*) were found in two patients each. These results suggest that SD is a genetically heterogeneous group. Four novel variants were found among the eighteen identified variants, which would be helpful in expanding the genotype–phenotype correlations.

In previous studies that attempted to diagnose patients with a suspected SD using NGS, the diagnostic yield was reported to vary widely depending on the genes included in the NGS panel, and the criteria used for selecting the patients. Zhang et al. (2015) performed a study in 82 patients with a suspected SD using an NGS panel containing 61 genes, which were selected according to the nosology and classification of genetic skeletal disorders in 2010. They found mutations in 13 different genes in 44 out of 82 patients, and reported a diagnostic yield of 54%. Polla et al. (2015) executed an NGS panel consisting of 309 genes in 69 patients with a suspected SD and reported a diagnostic yield of 18.8%. Bae et al. (2016) carried out the targeted exome sequencing (TES) for 255 genes in 185 patients with SD. Pathogenic variants were detected in 74% (71 out of 96) of the patients with an assured clinical diagnosis, and 20.3% (13 out of 64) of the patients with uncertain clinical diagnoses were detected in TES.

In our study, the total diagnostic yield was 51.6%. However, the diagnosis rates differed among the patient categories according to their clinical characteristics. The diagnostic yield was very high (11 out of 12, 91.6%) in patients with radiologic evidence that could clinically suggest SD (categories 1 and 2). Patients with a suspected familial SD (Categories 1 and 3) also showed a high diagnosis rate of 70% (7 out of 10). In contrast, the diagnostic yield for categories 4–6 was 31.2% (5 out of 16). Therefore, clinical phenotyping would still be important for a higher diagnostic yield of the NGS-based analysis. Among the patients in categories 1–3 in this study, pathogenic variants were found in genes such as *COL2A1, COMP*, and *MATN3*, which are the causative genes of type II collagenopathy and multiple epiphyseal dysplasia (MED). Although SD is a genetically heterogeneous group, type II collagenopathy or MED has been reported relatively frequently in most studies. In addition, patients with genetic mutations in *ACAN, PHEX, TRPV4, FBN1, and FGFR3* have also often been observed (Lazarus et al., 2014; Polla et al., 2015; Bae et al., 2016; Barat-Houari et al., 2016; Sentchordi-Montane et al., 2018; Uttarilli et al., 2019). Therefore, in these patients, a gene panel consisting of representative SD genes can reduce diagnostic costs.

In our study, 31.2% (5 out of 16) of the patients with skeletal involvement and other clinical manifestations including dysmorphism or MCA, and various degrees of DD/ID (categories 4–6) were able to obtain a definite diagnosis. Skeletal symptoms such as craniofacial abnormalities, scoliosis, kyphosis, or joint contractures are often accompanied with various syndromic DD/ID, and this should be considered by clinicians. Therefore, we included patients with DD/ID and various skeletal manifestations including scoliosis, kyphosis, multiple spinal fusion, syndactyly, clinodactyly, arachnodactyly, arthrogryposis, and DDH. Although the diagnosis rate was lower than that of patients with a high clinical suspicion of SD, the diagnosed patients in these categories had a rare syndromic SD such as spondylocarpotarsal fusion syndrome 1A (case 16), Loeys-Dietz syndrome 1 (case 17), Coffin–Siris syndrome (cases 21 and 22), and Yunis-Varon syndrome (case 30). In particular, the Yunis-Varon syndrome is extremely rare. Since its first report in 1980, fewer than 30 cases have been reported (Siddique et al., 2019; Wright et al., 2020). This is the first case reported in South Korea. It is difficult for clinicians to suspect these extremely rare diseases based on the clinical features or radiographic evidence, and other concomitant symptoms might not suggest SD. Therefore, NGS-based gene analysis might be helpful in increasing the diagnostic yield and expanding the knowledge regarding the clinical phenotypes for these rare diseases.

In this study, 43.7% (7 out of 16) of the diagnosed patients had a confirmed genetic diagnosis at under 3 years of age. For SD patients, especially those with other symptoms, definite diagnosis at an early age through genetic analysis could help in predicting undiscovered symptoms and in managing SD patients throughout their life. It is also important to understand the pathophysiology of SD, where symptoms change with the maturation of the skeletal system (Falardeau et al., 2017). Accurate genetic diagnosis facilitates family planning through genetic counseling and helps patients and their families accept the situation. In case 11, before all clinical symptoms of OI appeared, a genetic diagnosis was made, and complications such as fractures were sufficiently explained to the parents, a regular bisphosphonate infusion was started. In case 13, after a definite diagnosis through genetic study, we could find and correct a critical cardiac valve regurgitation and aortic aneurysm before the symptoms developed through surveillance for the involvement of other organs that may be accompanying the specific SD (Loeys-Dietz syndrome type 3). Two cases of Coffin-Siris syndrome diagnosed in this study were also evaluated and managed by a multidisciplinary team approach.

However, causative genetic variants were not identified in 15 patients (48.4%) in our cohort. For these patients, further interpretation of the NGS data, other than the 437 genes related to SD, could identify the causative genes. For instance, a patient with Cantu syndrome (case 23) caused by a heterozygous variant in *KCNJ8* (NM_004982.3: c.41T > G, p.Leu14Arg) was diagnosed through a further analysis of the NGS data. She presented facial dysmorphism (coarse face, depressed nasal bridge, hypertrichosis), umbilical hernia, growth retardation, and developmental delay since infancy. Although flaring of the metaphyseal plate was noted in the skeletal survey, initial analysis of the NGS data for SD did not identify any pathogenic variants. After Cantu syndrome was diagnosed by the interpretation of the expanded target genes, “reverse phenotyping” confirmed the diagnosis. Additional genetic investigations, including copy number variation (CNV) analysis, WES or WGS, are needed for other patients whose final diagnosis could not be made by the re-interpretation of the NGS data for the expanded target genes. In general, a considerable number of SD cases are known to be single gene disorders rather than disorders caused by CNVs such as duplications or microdeletions, and CNV has been studied more in DD/ID and in autism-spectrum disorders. However, some SDs have recently been reported because of the total deletion of genes included in the microdeletion of specific chromosomal loci (van Dijk et al., 2010; Jennes et al., 2011; Su et al., 2015; Puvabanditsin et al., 2018). In the third-generation sequencing, using CNV calling algorithms, it is possible to detect several CNVs in the NGS data; however, their sensitivity is generally lower than that of chromosomal microarray (CMA) and multiplex ligation-dependent probe amplification (MLPA). It is thus necessary to confirm with CMA or MLPA when CNV is suspected in NGS or WES. Furthermore, there is a possibility of finding new candidate genes and expanding the phenotypes of SD when WES or WGS is performed in these patients. In fact, with the development of the NGS technology including WES or WGS, more genes responsible for SD were identified, and 226 causative genes were included in the 2010 revision of the nosology and classification of genetic skeletal disorders (Warman et al., 2011), and this number was increased to 364 in 2015 (Bonafe et al., 2015) and 437 in 2019 (Mortier et al., 2019). In the newest version of the nosology of genetic skeletal disorders, compared with the previous version, pathogenic variants of 437 disease-causing genes have been found in 92% (425/461) of all disorders. A study on a large cohort in India has proposed that several genes not included in the nosology of genetic skeletal disorders 2019 are candidate genes for SD based on WES in patients with a suspected SD (Shaheen et al., 2016; Maddirevula et al., 2018; Uttarilli et al., 2019).

In conclusion, we performed a targeted NGS, including all 437 causative genes listed in the nosology of skeletal dysplasia published in 2019 (Mortier et al., 2019), in 31 patients with a suspected SD. We reported four novel variants, atypical clinical symptoms of known diseases, and extremely rare disease entities of SD to help expand the genotype–phenotype correlation of SD. To increase the diagnostic yield of suspected SD patients, it is important to categorize patients according to their clinical characteristics and to use the targeted NGS or WES appropriately. The limitation of this study is that this single-center study was carried out for a relatively short period of time, and the number of patients was not sufficient to demonstrate consistent characteristics or the genotype–phenotype correlations of patients in each category. Further research is thus needed to properly classify patients according to their clinical characteristics, family history, and radiological evidence to develop an applicable NGS panel based on patient classification, or to determine which patients need WES or WGS.

## Data Availability Statement

The datasets presented in this study can be found in online repositories. The names of the repository/repositories and accession number(s) can be found below: NCBI SRA, BioProject ID PRJNA718975, https://www.ncbi.nlm.nih.gov/sra/PRJNA718975.

## Ethics Statement

The studies involving human participants were reviewed and approved by the Institutional Review Board of Ajou University Hospital (AJIRB-BMR-GEN-20-519). Written informed consent to participate in this study was provided by the participants’ legal guardian/next of kin. Written informed consent was obtained from the individual(s), and minor(s)’ legal guardian/next of kin, for the publication of any potentially identifiable images or data included in this article.

## Author Contributions

SK analyzed and interpreted the data, and prepared and corrected the manuscript. S-ML, J-MC, and J-HJ reviewed the analysis of genetic information, and involved in the revision of the manuscript. HK, J-TK, and JC enrolled the participants and analyzed their clinical or radiological data. YS designed the study and approved the submitted version of the manuscript. All authors read and approved the final manuscript.

## Conflict of Interest

The authors declare that the research was conducted in the absence of any commercial or financial relationships that could be construed as a potential conflict of interest.

## Footnotes

1 http://browser.1000genomes.org/

2 http://evs.gs.washington.edu/EVS/

3 http://gnomad.broadinstitute.org/

4 http://coda.nih.go.kr/coda/KRGDB/index.jsp

5 http://sift.jcvi.org/

6 http://genetics.bwh.harvard.edu/pph2/

7 http://www.mutationtaster.org/
